# Incidental discovery of a giant ovarian cystadenoma

**DOI:** 10.1016/j.amsu.2022.104698

**Published:** 2022-09-15

**Authors:** Bouali Mounir, Elwassi anas, Eddaoudi Yassine, El Bakouri Abdelilah, El Hattabi Khalid, Fatimazahra Bensardi, Fadil Abdelaziz

**Affiliations:** a Visceral Surgery Emergency Department P35 , University Hospital Center Ibn Rochd, Casablanca, Morocco; b Faculty of Medicine and Pharmacy, Hassan II University, Casablanca, Morocco

**Keywords:** Serous cystadenoma, Giant ovarian tumor, Strangulated umbilical hernia

## Abstract

**Introduction:**

Serous cystadenomas account for approximately 25% of benign ovarian tumors in patients of childbearing age. Their growth is insidious, and the diagnosis can be difficult as they are often asymptomatic, Patients with serous cystadenoma often experience symptoms only if the lesion is twisted or has a mass effect because of its size.This was the case in our patient, whose cough and low back pain prompted her To consult a doctor, which led to the definitive diagnosis and treatment.

**Materials and methods:**

We report a case of a patient admitted for strangulated umbilical hernia with fortuitous discovery of a giant ovarian mass in the P35 visceral emergency department at the CHU ibn rochd hospital in Casablanca, Morocco.

**Results:**

the patient were operated in the emergency room, approached by laparotomy with the exploration we found umbilical hernia with a 6 cm long neck and necrotic bowel content a left latero-uterine mass of 40 cm of solid-cystic aspect and tube and right ovary without abnormalities and uterus of normal size the patient had an Segmental resection of 10 cm at 2.60 m from the ADJ and 1 m from the JIC with T-T grelo-grelic anastomosis and a left adnexectomy with a left latero-uterine mass of 45 cm and Epipoic and parietal peritoneum biopsy and Examination of the patient's spicemen showed serous cystadenoma weighing 10 kg and measuring 30 × 36*20 cm adjoining a tubular formation measuring 11 × 10*5 cm with bowel resection showed ischemic and hemorrhagic necrosis related to the occlusion with acellular ascites fluid.

**Discussion:**

Very Cystadenomas There are 2 types: Pleudomucinous cystadenomas or mucoid cysts, These are the most frequent neoplastic cysts of the ovary which are, in general, lumpy, multilocular, producing a gelatinous substance [8]. Their consistency is variable, some taut and firm, others semi-solid, spongy, thick, hollowed out “honeycomb” parts, which contain thick, stringy mucus. The coloration is variable: grayish white when the wall is thick, translucent in some places, with yellowish or white reflections, blue-black or reddish if there is spilled blood, grayish if there is cholesterol. on the other hand Serous cystadenomas or papillary cysts A little less frequent than mucoid cysts. Usually unilocular or paucicular, round or relatively flat, they contain protein-rich serous fluid. The coloration varies according to the content and thickness of the wall: light yellow or brown if the wall is thin, purplish red if there is blood, greyish white if the wall is thick. Consistency clearly fluctuates on the whole but can be hard in places, semi-solid, if there are abundant vegetations inside.

**Conclusion:**

Very large tumors have become curiosities in industrialized countries where the health care system is well developed. On the other hand, they are not rare in developing countries. The delay in diagnosis is most often due to the patient herself who does not consult out of ignorance or refusal of her pathology. But it can happen, and this is serious.

## Highlights

• The largest tumors found in the human species are essentially represented by human species are essentially represented by ovarian tumors.
• Its diagnosis is still very difficult.
• The treatment Serous cystadenomas geant is in most cases surgical.
• The diagnosis Serous cystadenomas is histological.

## 1 Introduction

The largest tumors found in the human species are essentially represented by ovarian tumors.

The development of the health care system and technologies have reduced the frequency of these large tumors.

However, they have not entirely disappeared for se**veral reasons: The signs of call are discreet for a long time are not always the same.** And The first signs, which appear with the increase in size of the tumor, are not very specific and can mislead a doctor who is not very familiar with the disease. and can mislead an unwary doctor who would make the mistake of not performing a gynecological examination. This gynecological examination would have to suspect a pelvic pathology.

The diagnosis can thus be delayed, leaving time for the cyst to take on significant dimensions and reach a gigantic size.

## 2 Patient and observation

**Fig. 1:**
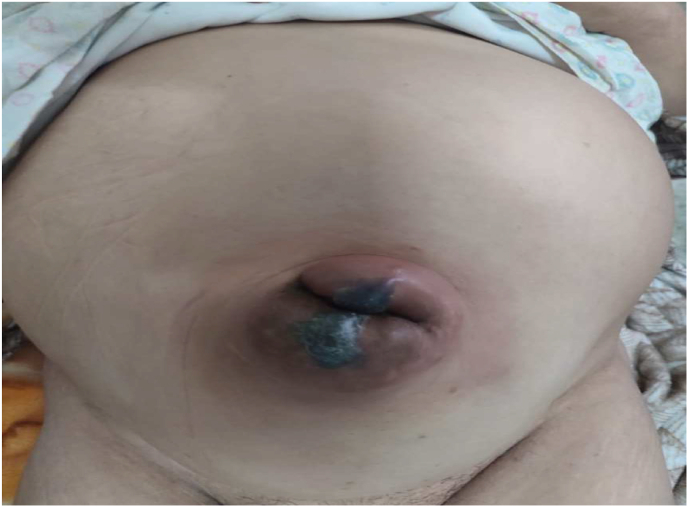
Strangulated umbilical hernia with inflammatory sign opposite.

She is a 63 years old patient having as medical history: operated for uterine myoma 20 years ago whose history of the disease goes back to 2 years by the appearance of a painless umbilical tumefaction reducible impulsive to coughing, complicated 4 days ago by becoming painful irreducible not impulsive to coughing associated with an occlusive syndrome made of stop of matter and gas with food vomiting, all evolving in a context of apyrexia and alteration of the general state.with clinical examination: conscious patient stable on the hemodynamic and respiratory plan The examination noted a distended abdomen, hypertympanic, Presence of a pfannestiel scar, Presence of a strangulated umbilical hernia with inflammatory sign opposite (Fig. 1)., the rectal ampulla was empty and The rest of the somatic examination: without particularitiese The biological work-up revealed a hb 12,7 g/dL; hyperleukocytosis with predominantly PNN at 14 000 elements/mm3, CRP was elevated to 350mg/L Albumin: 35 g/L, renal function was normal urea 5 mmol/L creatinemia 9 mg/L, unprepared abdomen in the standing position, which revealed a hydroaerobic level of the greaves.

**Fig. 2:**
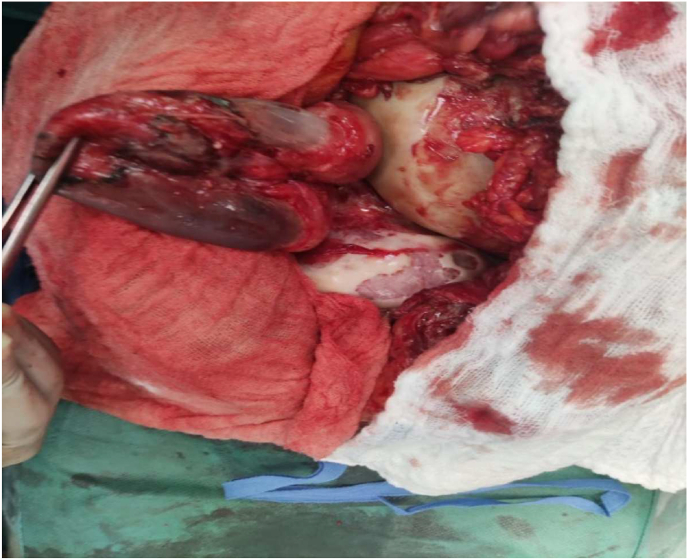
Necrotic bowel.

**Fig. 3:**
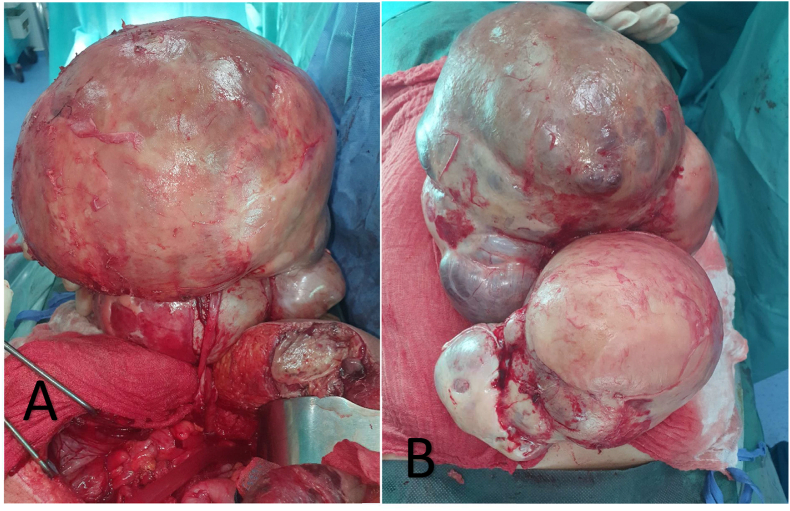
Intraoperative view showing; ovarian mass before adnexectomy.

The patient were operated in the emergency room, approached by laparotomy with the exploration we found umbilical hernia with a 6 cm long neck and necrotic bowel content (Fig. 2), a left latero-uterine mass of 40 cm of solid-cystic aspect and tube and right ovary (Fig. 3): without abnormalities and uterus of normal size (Fig. 3) the patient had an Segmental resection of 10 cm at 2.60 m from the ADJ and 1 m from the JIC with T-T grelo-grelic anastomosis and a left adnexectomy with a left latero-uterine mass of 45 cm and Epipoic and parietal peritoneum biopsy.

**Fig. 4:**
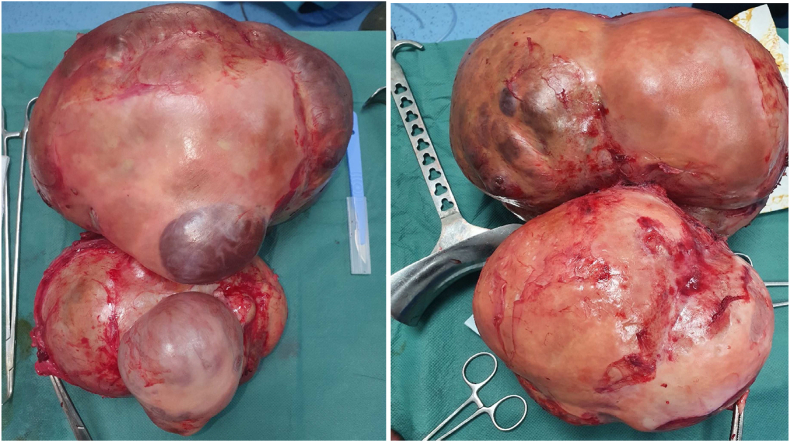
After operative view showing.

**Fig. 5:**
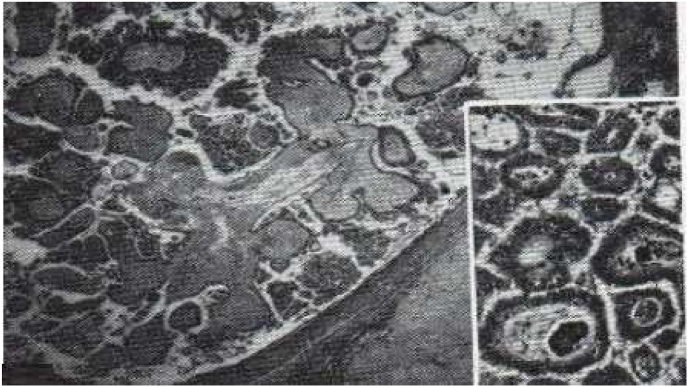
Serous cystadenoma with globular vegetations with abundant connective tissue axis and branching papillary vegetations, poor in connective tissue; note on the right, the two calcospherites in the axis of the papillary vegetation; also note the clear limitation of the lesion.

Examination of the patient's spicemen (Fig. 5) showed serous cystadenoma weighing 10 kg and measuring 30 × 36*20 cm adjoining a tubular formation measuring 11 × 10*5 cm with bowel resection showed ischemic and hemorrhagic necrosis related to the occlusion with acellular ascites fluid (Fig. 4)

Patient follow-up was every 15 days with a complete clinical examination with primary care of the surgical wound with instructions to change the dressing every 2 days and take antibiotics, anticoagulants and antlagics for 15 days and to avoid carrying heavy loads until complete healing.

The postoperative follow-up was simple and the patient was declared discharged on day 7 postoperatively.

The surgical procedure was performed on a scheduled date with a correct pre-anesthetic assessment; the procedure was performed by an assistant professor in general surgery and two residents in the same specialty.

The operation was performed in the operating room of the P35 visceral emergency department at the CHU ibn rochd hospital in Casablanca, Morocco the patient was satisfied with the intervention and the improvement of his health in the short and long term.

The work has been reported in line with the SCARE 2020 criteria [1].

## 3 Discussion

Cystadenomas There are 2 types: Pleudomucinous cystadenomas or mucoid cysts, These are the most frequent neoplastic cysts of the ovary which are, in general, lumpy, multilocular, producing a gelatinous substance [2]. Their consistency is variable, some taut and firm, others semi-solid, spongy, thick, hollowed out “honeycomb” parts, which contain thick, stringy mucus. The coloration is variable: grayish white when the wall is thick, translucent in some places, with yellowish or white reflections, blue-black or reddish if there is spilled blood, grayish if there is cholesterol. on the other hand Serous cystadenomas or papillary cysts A little less frequent than mucoid cysts. Usually unilocular or paucicular, round or relatively flat, they contain protein-rich serous fluid. The coloration varies according to the content and thickness of the wall: light yellow or brown if the wall is thin, purplish red if there is blood, greyish white if the wall is thick. Consistency clearly fluctuates on the whole but can be hard in places, semi-solid, if there are abundant vegetations inside [3,4].

Serous cystadenoma or Serous cyst of the ovary is a benign epithelial tumor of the ovary accounting for 20–25% of all benign tumors of the ovary. It grows on the coelomic epithelium (the epithelium covering the ovary) and accounts for 50% of all epithelial tumors [[5], [6], [7], [8], [9]].

The epithelium lining the cystic cavities is cubic, uni-stratified, occasionally ciliated. The axis of these vegetations contains calcospherites, in varying numbers. The serous fluid, sometimes mucoid, is clear, yellowish or hemorrhagic. Cystadenoma, uni or plurilocular, may cause only a slight increase in size. A tumor variety specific to the ovary, these tumors are situated between morphologically benign lesions and malignant tumors. They should be singled out because of their frequency, their age of occurrence (lower than that of malignant tumors) and above all their excellent prognosis compared to that of malignant tumors. Macroscopically, they are usually cystic tumors with endocystic and sometimes exocystic vegetations [10].

Serous cysts of the ovary preferably appear before the age of 40, but in fact they can be observed at any age. Usually they are benign tumors, but some are borderline or malignant.

The serous cyst of the ovary is classically unilocular, but sometimes it is multilocular, measuring on average 10 cm in diameter; in some cases they can reach the size of 30–50 cm, with a volume varying from a few cubic centimetres to several tens of liters. Ovarian serous cysts are bilateral in 12–20% of cases [11,12].

The content of the cyst varies according to the nature of the cyst, sometimes a gelatinous substance, if it is mainly a mucoid cyst, sometimes a hematic liquid, homogeneous or mixed with yellowish and clear debris, if it is a serous cyst, and sometimes a blackish liquid containing hairs, which is found in the dermoid cyst and represents most cases [13,14].

In benign forms of serous cysts, the external surface is regular, smooth, shiny and rich in blood vessels; the cyst wall is thin, less than 3 mm thick, and sometimes translucent.Its contents are fluid, serous, like rock water or citrine and sometimes viscous [14,15].

The internal surface of the cyst is smooth and regular, but sometimes small endophytic vegetations in the form of papillary structures and also some fine septa are present in a localized manner [16].

The epithelium lining the inner surface of the serous cyst is composed of tubular, regular, unistratified, cubic or sometimes cylindrical cells with basal nuclei. Some cells are clear and ciliated. This epithelium is comparable in many respects to the internal epithelium of the uterine tube.

Malignant forms of serous tumors of the ovary represent 40–50% of all malignant tumors of the ovary. In fact, among serous ovarian tumors: 50–70% are benign; 10–20% are bilateral; 10–15% are borderline tumors, generally with a good prognosis; 25–30% are malignant tumors (serous cystadenocarcinomas) [17].

These types of lesions are most often asymptomatic. When they are large, they are likely to cause pain, abdominal transit disorders, increased abdominal perimeter or dysuria [[2], [7], [8], [9], [18], [19], [20]]. They may sometimes manifest as metrorrhagia or feminization, which are thought to be secondary to the hyperestrogenism induced by the tumor [[2], [3], [4], [10], [11], [12], [16], [17], [20], [21], [22], [23], [24], [25], [26], [27], [28], [29], [30], [31], [32], [33]]. The mechanism underlying this hyperestrogenism would be a hypersecretion of hormone by the tumor itself [[3], [4], [17], [32], [33], [34]].

In such a context, it is necessary to recall the interest of an extemporaneous examination in the management of an ovarian tumor suspected of malignancy. This should be requested if the tumor presents worrying macroscopic characteristics (vegetations, thick septa, suspicious intracystic fluid) and if the results of this examination are likely to modify the operative strategy. If this request for an extemporaneous examination is anticipated before the operation, the patient must be informed of the possible modifications that its conclusions may entail concerning the operative strategy (laparoconversion, bilateral hysterosalpingo-oophorectomy, lomboaortic and pelvic lymphadenectomies) [29,30].

However, in cases where extemporaneous examination is not feasible or uncertain, the laparoscopic appearance described in this article should prompt the surgeon to defer extensive first-line surgical treatment [26].

Laparoscopy can show the types of cyst, which is translucent if it is serous, bumpy and greenish if it is mucoid - can specify the existence or absence of exocystic vegetations - allows other pathologies to be eliminated, in particular, ectopic pregnancy (EP), non-ovarian genital masses and certain pseudocystic formations [20,21].

The procedure to be proposed in the case of a small anechogenous serous cyst is treatment by laparoscopic puncture, which persists despite hormonal blockade. On the other hand, small cysts less than 8 cm in diameter should be left to develop, because if it is a functional cyst, it will disappear with or without medical treatment after 3 months [27,28].

In the case of unilateral ovarian cysts in postmenopausal women, adnexectomy should be performed. In the case of large cysts, efforts should be made to retain as much ovarian parenchyma as possible, for which oophorectomy or adnexectomy by laparotomy or laparoscopy are theoretically indicated [22].(12) [24](14)

There are no macroscopic criteria to differentiate them from benign papillary cystadenomas on the one hand and malignant tumors or cystadenocarcinomas on the other. Histologically, the cells lining the cyst wall and the papillae reflect the proliferative nature of the lesion [35].

There are pluristratifications of the epithelial lining, clumps of epithelial cells shedding into the cyst lumen, cytonuclear atypia, and mitoses. There is no infiltration of the stroma and The prognosis of serous tumors at the limit of malignancy is very good [10].

## 4 Conclusion

Very large tumors have become curiosities in industrialized countries where the health care system is well developed. On the other hand, they are not rare in developing countries. The delay in diagnosis is most often due to the patient herself who does not consult out of ignorance or refusal of her pathology. But it can happen, and this is serious, that this delay is caused by the doctor. Indeed, The doctor may falsely reassure the patient because of the lack of a good clinical examination, which is very often sufficient to evoke the diagnosis in the face of imprecise abdominal signs.

## Ethical approval

I declare on my honor that the ethical approval has been exempted by my establishment.

## Please state any sources of funding for your research

None.

## Author contribution

El wassi Anas: Corresponding author writing the paper and operating surgeon.

Eddaoudi Yassine: Corresponding author writing the paper and operating surgeon.

Bouali Mounir: writing the paper and operating surgeon.

El Bakouri Abdelilah: study concept; El Hattabi Khalid: study concept; Bensardi Fatimazahra: study concept.

Fadil Abdelaziz: correction of the paper.

## Registration of research studies

Researchregistry2464.

## Guarantor

DR WAS EDD.

## Consent

Written informed consent for publication of their clinical details and/or clinical images was obtained from the patient.

## Provenance and peer review

Not commissioned, externally peer-reviewed.

## conflict of interest

The authors declare having no conflicts of interest for this article.
